# Response to Moore and others (2010) and Mehta (2010)

**DOI:** 10.4269/ajtmh.2011.10-0584a

**Published:** 2011-02-04

**Authors:** Adam L. Cohen, Daniel R. Feikin, Orin S. Levine, Cynthia G. Whitney

**Affiliations:** Division of Bacterial Diseases; National Center for Immunization and Respiratory Diseases; Centers for Disease Control and Prevention; Atlanta, Georgia; E-mail: dvj1@cdc.gov; Division of Preparedness and Emerging Infections; National Center for Emerging Zoonoses and Infectious Diseases; Centers for Disease Control and Prevention; Atlanta, Georgia; International Vaccine Access Center; Department of International Health; Johns Hopkins Bloomberg School of Public Health; Baltimore, Maryland; International Vaccine Access Center; Department of International Health; Johns Hopkins Bloomberg School of Public Health; Baltimore, Maryland; Division of Bacterial Diseases; National Center for Immunization and Respiratory Diseases; Center for Disease Control and Prevention; Atlanta, Georgia

Dear Sir:

We read with great interest the article on pneumococcal serotyping in Laos by Moore and others^1^ and the accompanying editorial by Mehta^2^ in the September 2010 issue of *The American Journal of Tropical Medicine and Hygiene*. We commend Moore on describing the pneumococcal serotype distribution in this country in Asia, a region with relatively few data on pneumococcal serotypes causing invasive disease.^3^ However, we draw different conclusions from the data than Mehta did in his editorial, and propose that these data support the use of currently available pneumococcal conjugate vaccines in Asia now, rather than further delays. What is missing in much of the developing world right now is high-quality surveillance rather than high-quality vaccines.

An estimated 800,000 children died of pneumococcal disease in 2000, with much of the burden in Asia.^4^ The World Health Organization recommends that all countries introduce pneumococcal conjugate vaccine into their routine childhood immunization systems.^5^ Countries that newly introduce pneumococcal conjugate vaccines into their routine childhood immunization systems will not introduce the 7-valent pneumococcal conjugate vaccine (PCV7), which is soon to be phased out of production, but rather the currently available 10- or 13-valent pneumococcal conjugate vaccines. Although more than 90 pneumococcal serotypes have been identified, a recent review article on serotype distribution worldwide found that serotypes included in the 10- and 13-valent pneumococcal conjugate vaccine formulations cause at least three-quarters of disease in all regions, including Asia.^3^ Indeed, Moore reported that while PCV7 includes serotypes causing less than half of invasive pneumococcal disease (IPD) in Laos, the 10- and 13-valent pneumococcal vaccines include the majority of serotypes causing disease (73% and 73%, respectively).^1^

The editorial's suggestion that pneumococcal bacteria and influenza viruses are similar is incorrect. Although pneumococcal serotypes causing disease in a community can vary a little in their individual incidence from year to year, especially if outbreaks occur, the bacteria do not continually “drift” over time like influenza strains; therefore, pneumococcal vaccines do not require annual updates. Decreases in invasive pneumococcal disease caused by serotypes in PCV7 among children of the age targeted for vaccination have been rapid, substantial, and sustained. Despite variable increases in rates of IPD caused by serotypes not in PCV7, overall reductions in rates of IPD following introduction of PCV7 in children have been consistently observed in multiple countries.^6^–^8^ The editorial suggests that serotypes 1 and 5 might be selected by use of PCV7; however, increases in disease caused by these serotypes has not been seen in countries that have introduced PCV7 and substantial outbreaks of each have been observed in the absence of vaccination.^9^ More importantly, both serotypes 1 and 5 are included in 10- and 13-valent vaccines and, as such, vaccination should make these serotypes less, not more, likely to cause disease.

We agree about the urgent need for ongoing surveillance to monitor changes in pneumococcal epidemiology that can help policymakers make informed decisions regarding their pneumococcal vaccine programs. In interpreting surveillance data, the public health impact of vaccines will be best reflected by monitoring changes in the overall rate of IPD, rather than focusing more narrowly on percent increases in non-vaccine types. On the basis of all available data, currently available pneumococcal conjugate vaccines cover about three-quarters of all the serotypes causing disease in young children. Global surveillance for IPD, on the other hand, does not yet cover three-quarters of the places where we should have it.

Perhaps it is time to shift the focus from expanding the serotypes in the vaccine to expanding access to the vaccine for children in countries like Laos, and assuring that the surveillance systems are in place to monitor the vaccine's effects. Mehta states that countries should find “the right pneumococcal vaccine for the time and place.” We agree. With 10- and 13-valent vaccines available right now through the GAVI Alliance for countries like Laos, the data in the paper by Moore suggest that the right vaccines are here, and the right time is now.
